# Mating system, morphological and genetic evidence endorse clonality as an essential reproductive mode in *Daphnopsis filipedunculata* (Thymelaeaceae), a dioecious and endemic species from the Amazon

**DOI:** 10.1093/aobpla/plae048

**Published:** 2024-09-10

**Authors:** Carolina da Silva Carvalho, Lucas Erickson Nascimento da Costa, Bárbara Simões Santos Leal, Kleber Resende Silva, Adriano Valentin-Silva, Ana Carolina Galindo Costa, Lourival Tyski, Fernando Marino Gomes dos Santos, Mauricio Takashi Coutinho Watanabe

**Affiliations:** Instituto Tecnologico Vale—Desenvolvimento Sustentável, Rua Boaventura da Silva 955, Belém, Pará 66055-090, Brazil; Instituto Tecnologico Vale—Desenvolvimento Sustentável, Rua Boaventura da Silva 955, Belém, Pará 66055-090, Brazil; Instituto Tecnologico Vale—Desenvolvimento Sustentável, Rua Boaventura da Silva 955, Belém, Pará 66055-090, Brazil; Instituto Tecnologico Vale—Desenvolvimento Sustentável, Rua Boaventura da Silva 955, Belém, Pará 66055-090, Brazil; Instituto Tecnologico Vale—Desenvolvimento Sustentável, Rua Boaventura da Silva 955, Belém, Pará 66055-090, Brazil; Instituto Tecnologico Vale—Desenvolvimento Sustentável, Rua Boaventura da Silva 955, Belém, Pará 66055-090, Brazil; Plano de Gestão da Biodiversidade de Carajás (PGBio), Gerência de Estudos Técnicos de Longo Prazo, Vale S.A., Rodovia Raimundo Mascarenhas Km 26, s/n, Núcleo Urbano de Carajás, Parauapebas, Pará 64516-000, Brazil; Gerência de Licenciamento Ambiental, Vale S/A, Alameda Oscar Niemeyer 132—Edifício Concórdia, Nova Lima, Minas Gerais 34006-049, Brazil; Instituto Tecnologico Vale—Desenvolvimento Sustentável, Rua Boaventura da Silva 955, Belém, Pará 66055-090, Brazil

**Keywords:** Campo rupestre on canga, clonality, *Daphnopsis filipedunculata*, dioecy, endemic species, root anatomy, sexual reproduction, vegetative propagation

## Abstract

*Background and Aims:* Clonality is characterized by the formation of independent individuals of the same genotype that are capable of reproducing and propagating vegetatively. Although clonality is an important mechanism that facilitates the persistence of a population, its extensive use can lead to negative impacts on sexual reproduction due to trade-offs in the investment of resources. Therefore, studies on the sexual reproduction of species that exhibit clonality can provide information about resilience to environmental changes, information about fecundity, the risk of the absence of pollinators and the ability to persist in unfavourable conditions and to successfully occupy new areas. Here, we investigated the role of clonal propagation and sexual reproduction in *Daphnopsis filipedunculata* (Thymelaeaceae), a dioecious species distributed only in Serra dos Carajás. *Methods:* We evaluated the extent of clonality in this species using molecular tools and anatomical analyses of the underground system responsible for developing new ramets. Furthermore, we analysed the sexual system and its contribution to reproductive success through morphometric analyses of floral types and pollination experiments in the field. *Key Results:* Overall, we found that clonal propagation plays an important role in maintaining the population of *D. filipedunculata*. Specifically, we demonstrated that this species presents functional male and female plants, indicating that *D. filipedunculata* is an obligate xenogamous species but has low reproductive success. We also showed that clonal vegetative propagation is the main form of asexual reproduction in this species, with roots responsible for clonal growth. Finally, our results indicated that this species presents an intermediate phalanx–guerrilla clonal architecture. *Conclusions:* Our study provides the first insights into sexual reproduction and clonal propagation in *D. filipedunculata* and can inform management practices, conservation and the restoration of endemic species.

## Teaser

In this study, we integrate molecular tools, anatomical and morphometric analyses and pollination experiments to investigate the importance of clonal propagation and sexual reproduction in *Daphnopsis filipedunculata* population, an endemic species from the Eastern Brazilian Amazon. We found that clonal propagation plays an important role in the *D. filipedunculata* population. Specifically, we demonstrated that this species presents female and functional male phenotypes, indicating that *D. filipedunculata* is an obligate xenogamous species but has low reproductive success. We also showed that clonal propagation is the main mode of reproduction in *D. filipedunculata* and that roots are the organ responsible for clonal growth. Finally, our results indicated that *D. filipedunculata* presents an intermediate phalanx–guerrilla clonal architecture. Our findings have important implications for the conservation of this endangered, highly endemic species.

## Introduction

Clonality is characterized by the formation of independent individuals of the same genotype capable of reproducing and propagating vegetatively (Harper 1977); this form of asexual reproduction is common in 80 % of angiosperm species (Klimes *et al.* 1997). Clonal growth can be accomplished by several morphological organs (i.e. rhizome, stolons, roots), which are not functionally equivalent and can be triggered by different drivers (Klimešová *et al.* 2017; Herben and Klimešová 2020). Most clonal plants emit ramets from underground organs—roots and/or stems, which can be distinguished anatomically (see Klimešová *et al.* 2019). Compared to the stems, the roots are deeper, protecting the buds that form the new ramets and can reach greater distances (Klimešová *et al.* 2019), interfering with the distance between the genets. Furthermore, roots are directly associated with nutrient acquisition and root sprouting constitutes an independent route to clonal propagation (Klimešová *et al.* 2017; Herben and Klimešová 2020). Contrary to stem-based clonality that does not evolve to promote species occurrence in disturbed habitats, clonal growth throughout root sprouting is an important trait for response to disturbance (Klimešová *et al.* 2017; Herben and Klimešová 2020).

Furthermore, clonal growth organs can display different spatial arrangements of ramets (i.e. clonal architecture). For instance, tillers, bulbs and rhizomes tend to generate ramets relatively close to the parent, whereas stolons and runners emit ramets placed at greater distances from their parents (Barrett 2015). It characterizes two contrasting ecological strategies: (a) the phalanx strategy, which results in a close aggregation of ramets, usually packed around the parental shoot and (b) the guerrilla strategy, which is characterized by an extensive intermingling of ramets from different genets (Ye *et al.* 2006; Barret *et al.* 2015). The guerrilla strategy is associated with resource-heterogeneous or disturbed habitats, while phalanx strategy is more related to homogeneous and less disturbed habitats (Ye *et al.* 2006). Regarding its importance, more detailed analyses of belowground organs responsible for clonality should not be overlooked because it may have a direct impact on clonal architecture and consequently on the benefits and disadvantages of clonality (Charpentier 2001; Thomas and Hay 2010; Klimešová *et al.* 2017).

An increasing number of studies have shown that asexual reproduction through clonal propagation has significant benefits for population growth (Klimes *et al.* 1997; Xiao *et al.* 2011; Chen *et al.* 2015). As clonality requires a lower investment when compared to reproduction via seeds, it can boost population growth, favouring species persistence when sexual reproduction is restricted (Barrett 2015; Klimešová *et al.* 2021). Moreover, physiological integration amongst ramets can be advantageous due to the sharing of multiple resources (e.g. water, carbohydrates and mineral nutrients) and information (signalling molecules) as well as the potential division of labour within the genet (Callaghan 1984; Dong 1996; reviewed by Liu *et al.* 2016). It can reduce the likelihood of the death of genets, allowing them to cope with environmental heterogeneity (Dong 1996; Liu *et al.* 2016).

Although clonality is an important mechanism to facilitate population persistence regardless of sexual reproduction (Barrett 2015; Hu *et al.* 2017), extensive clonality may increase susceptibility to diseases and other disturbances due to a lack of genetic variation (Lei 2010) and affect sexual reproduction in a manner that decreases plant fitness (Barrett 2015). Trade-offs between clonality and sexual reproduction are mainly due to (a) resource investment, as clonal reproduction may limit resource allocation to flowering and seed production (Barrett 2015; Hewitt 2020), and (b) the effects of clonal architecture on mating availability (Charpentier 2001; Honnay and Jacquemyn 2008; Thomas and Hay 2010; Barrett 2015). In fact, low fruit sets and overall lower reproductive success have been reported in clonal species (Sydes and Peakall 1998; Faria *et al.* 2006; Herben *et al.* 2012; Franklin *et al.* 2021). Clonality may enhance geitonogamy, increasing fitness costs in self-compatible species due to higher selfing rates and reduction of genetic diversity (Ushimaru and Kikuzawa 1999; Honnay and Jacquemyn 2008; Dering *et al.* 2015). This selective pressure imposed by geitonogamy on the mating system can explain the correlated evolution of self-incompatibility and clonality (Charpentier 2001; Honnay and Jacquemyn 2008).

Nevertheless, self-incompatible, dioecious and other sexual polymorphic species can experience more severe consequences from clonal propagation, which, in some cases, leads to a disruption of sexual reproduction (Barrett 2015; Hu *et al.* 2017). First, clonality can lead to a sex-biased population, usually male-biased, due to the low reproductive costs often attributed to male over female plants (Delph 1999; Sinclair *et al.* 2012; Field *et al.* 2013; Khanduri *et al.* 2019). This might favour the occurrence of single-sex monoclonal patches where clonal propagation is the only reproductive mechanism, significantly reducing population viability (Honnay and Bossuyt 2005; Barrett 2015). Furthermore, various combinations of clonal architecture and clone size may have important implications for mating, fertility and persistence of self-incompatible and sexual polymorphic species (Barrett 2015). For example, spatially clustered ramets (phalanx growth form) are advantageous for optimizing resource capture and space occupation. However, this growth form is expected to decrease mate availability in self-incompatible species as clonal patches increase (Barrett 2015). Moreover, dioecious or self-incompatible species with a guerrilla growth form can benefit from clonality, which can help to maintain genetic diversity when combined with an outcrossing mating system (Vallejo-Marín and O’Brien 2006; Hu *et al.* 2017).

Plants have repeatedly evolved asexual reproduction in tandem with sexual reproduction (Klimes *et al.* 1997), a combined strategy proposed to ensure the transmission of well-adapted genes while simultaneously providing the genetic variability necessary to colonize new habitats and survive future environmental changes (Niklas and Cobb 2017). Investigating the multiple outcomes in the trade-off between sexual reproduction and vegetative propagation can provide relevant information about the resilience of species to environmental changes, such as fertility, the risk of pollination failure and the persistence of a species under unfavourable conditions or the ability to successfully occupy new sites (Sydes and Peakall 1998; Adam and Williams 2001; Butcher *et al.* 2011; Hu *et al.* 2017). These issues might be critical for threatened species, as extensive clonality in these species can have important implications for their conservation status (Sydes and Peakall 1998; Hu *et al.* 2017). Indeed, clonality can contribute to extinction debt, and the current distribution of a species might not reflect the ecological viability of its populations (Eriksson and Ehrlén 2001).

Thymelaeaceae is a family with several species with clonal reproduction that has received particular attention from researchers examining mating system evolution given its variable sexual expression (Herber 2003; Beaumont *et al.* 2006). Thymelaeaceae has approximately 800 species and is split into 2 subfamilies, Octolepidoideae and Thymelaeoideae (Rogers 2010), which mainly include hermaphroditic, dioecious and gynodioecious species (Herber 2003). Approximately one-third of the genera in Thymelaeoideae have unisexual flowers, and most of their species are sexually dimorphic (Beaumont *et al.* 2006). Most studies on sexual expression have been carried out with gynodioecious species of *Daphne* (Medrano *et al.* 2005; Alonso *et al.* 2007; Alonso and Herrera 2011; Shibata *et al.* 2018, 2021), *Gnidia* (Beaumont *et al.* 2006; Smith 2009) and *Pimelea* (Merrett 2007) or with *Thymelaea hirsuta*, an interesting species with a tetramorphic sexual system (Dommée *et al.* 1990, 1995; Shaltout and El-Keblawy 1992; El-Keblawy *et al.* 1996; Minuto *et al.* 2005); these species occur in Australian, Ethiopian and Palaeartic regions. Neotropical genus, such as *Daphnopsis* (the largest genus in the New World), have received less attention in studies of mating systems (Bullock 1985; Ramírez and Briceño 2021).

*Daphnopsis* is an exclusively dioecious genus that predominantly has morphologically unisexual flowers (organs of one sex are functional, whereas those of the other are deformed/modified or vestigial, *sensu*Mayer and Charlesworth 1992), especially species of the subgenus *Daphnopsis* (Nevling Jr. 1959, 1963). However, some plants of *D. americana* have flowers that appear to be functionally bisexual and produce fruits (Nevling Jr. 1959). According to this author, there are also reports of functionally male plants with flowers in which the ovary contains a relatively well-formed seed. In the subgenus *Neivira*, pistillate flowers usually have a staminode, and staminate flowers usually have a well-developed pistillode (Nevling Jr. 1959). However, there have been no further investigations into the seed production capacity of staminate flowers with developed pistillodes in this genus. *Daphnopsis filipedunculata* Nevling & Barringer (Thymelaeaceae) is a dioecious, rare and endangered species endemic to Brazil with distribution restricted to the Serra dos Carajás in the eastern Amazon (Nevling Jr. and Barringer 1993; Mota and Giulietti 2016; Watanabe *et al.* 2018; Rossi 2020). Although *D. filipedunculata* has been recorded as a dioecious species (Mota and Giulietti 2016; Watanabe *et al.* 2018), the specifics of its reproductive system have never been investigated. Previous studies have reported that some sites of occurrence of *D. filipedunculata* are formed by clumps of individuals, with some presenting clonal behaviour similar to other Thymelaeaceae species (Rogers 2009; Watanabe *et al.* 2018). Moreover, the currently monitored population exhibit a male-biased population structure with a low frequency of female plants (Watanabe *et al.* 2018).

Here, we investigated the importance of clonal propagation and sexual reproduction in *D. filipedunculata* population. We hypothesized that if, on the one hand, clonality is the dominant mechanism in *D. filipedunculata*, most individuals in the population will consist of the same genotype. On the other hand, predominantly sexual reproduction would result in most individuals presenting unique genotypes. To test these predictions, first, we investigated the presence and extent of clonality using genomic data to identify potential clones. In addition, we performed anatomical analyses to identify from which organ the new ramets develop and the occurrence of reserve substances. Since stem and root sprouting have different triggers, it might have different implications for the explanation of clonality patterns in *D. filipedunculata.* Second, we analysed the sexual system and its contribution to reproductive success. We performed morphometric analyses of the two floral types to assess the existence of sexual dimorphism. Through pollination experiments on both floral types, we verified whether staminate flowers with developed pistillodes act as functional males or as true hermaphrodites, potentially generating fruits and whether these flowers can perform self-fertilization or whether the species rely solely on outcrossing. Finally, we discussed the implications of the balance between sexual reproduction and vegetative propagation for the conservation of this treelet species.

## Materials and Methods

### Study area

**Figure 1.**
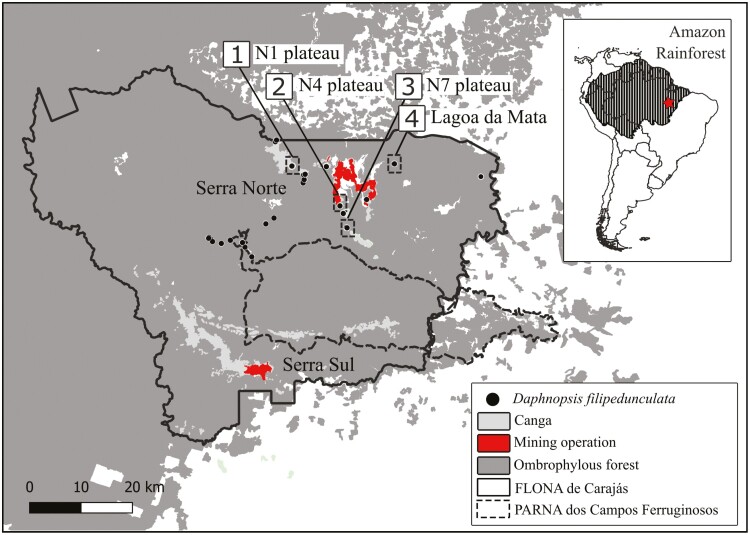
Study area, sampling sites and distribution range of *D. filipedunculata* in the FLONA de Carajás and PARNA dos Campos Ferruginosos, eastern Amazon, Brazil. Sampling sites are in the transition zone between ‘*cangas*’ and ombrophilous dense forest in the N1 plateau (1), Lagoa da Mata (2), N4 plateau (3) and N7 plateau (4).

The study was conducted in the Floresta Nacional de Carajás (FLONA de Carajás), a protected area with sustainable use of natural resources of 411.949 ha located in the Eastern Brazilian Amazon (IBAMA 2003; Fig. 1). FLONA Carajás is composed of ironstone outcrops (known as campo rupestre on ‘*canga*’), a unique ecosystem limited by iron concentration in the soils, nestled within ombrophilous dense or open forests (Fig. 1). *Daphnopsis filipedunculata*, a treelet species (1–8 m in hight), grows in the transition zone between the ‘*canga*’ ecosystem and the ombrophilous forest (Nevling Jr. and Barringer 1993), and more recently was recorded within the ombrophilous forest (Amplo Engenharia 2021). We carried out most of the experiments in the transition zone between the N1 plateau (‘*canga*’ ecosystem) and the ombrophilous forest (Fig. 1) to control for any differences due to environmental conditions. This is the area where the species was first recorded growing (Nevling Jr. and Barringer 1993) and presents a high abundance of individuals (personal communications). To increase the sample size for the experiments on the sexual system, we also sampled individuals from other transition zones that present similar environmental conditions (Lagoa da Mata, the N4 plateau and the N7 plateau, Fig. 1).

### Identification of clonemates using high-throughput sequencing

To determine potential clonal individuals, that is, those that present the same multilocus genotype, we covered the entire known distribution area of *D. filipedunculata* in the transition zone between the N1 plateau and the ombrophilous forest in 2021 (SISBIO collection permit N. 76784-1) and sampled 49 individuals, without delimiting a minimum distance for sampling. The sampled individuals had different sizes. There is no information on whether the height of the plants is related to the age of the individuals, but we found reproductive individuals measuring less than one metre. These samples corresponded to 70 % of the known individuals on the southern edge of the N1 plateau at that time, and their distance ranged from less than a metre to 247 m. From each individual, we collected leaflet samples for genotype analysis that were preserved in CTAB and stored at −20 °C until DNA extraction. The geographic location of each sample was recorded with a GPS device. Among the sampled individuals, 36 were classified as male plants, 3 as female plants and 10 as undetermined (due to a lack of reproductive structures).

Total DNA was extracted with the Qiagen DNeasy Plant Kit and quantified with the Qubit High Sensitivity Assay Kit (Invitrogen), and its degradation was assessed by electrophoresis in 1.2 % agarose gels. Samples were then shipped to Ecomol Consultoria (https://ecomolconsultoria.com.br/) for sequencing. We performed genotype-by-sequencing using the protocol described by Elshire *et al.* (2011), which consists of reducing genome complexity using restriction enzymes. Here, all samples were digested using the PstI enzyme. Then, the DNA fragments were ligated to adapters containing specific barcodes for each individual. The restriction-ligation products were pooled and enriched through PCR. The library was then sequenced with two lanes on an Illumina NovaSeq 6000 instrument using an SP 100 cycle (1 × 100 bp) kit.

We sequenced a mean of 2 530 789.7 reads per sample (min. 595 441 and max. 5 769 606 reads). The raw sequence reads were *de novo* assembled in ipyrad software (Eaton and Overcast 2020) using a pipeline for species without a reference genome, and 99.96 % of reads passed ipyrad filtering procedures. The *de novo* assembly resulted in a mean of 16 053 locus per sample (min. 7243 and max. 21 384). We set the analysis parameters as suggested by the authors, except for the following: clust_threshold = 0.95, filter_adapters = 2 (stricter), max_Indels_locus = 4. VCFTools was further used to obtain a final dataset of biallelic SNPs (--remove-indels, --max-alleles 2 and --min-alleles 2) without missing data (--max-missing 1) and with minimum allele frequency of 2 % (--maf 0.02) and a minimum depth of 10 (--minDP 10). We did not filter our dataset for one SNP per locus. However, our dataset presents a low mean number of SNPs per locus (= 1.23 SNPs/locus), thus it is unlikely to affect the genetic diversity results.

To identify potential clones, we used the ‘poppr’ R package (Kamvar *et al.* 2014), which creates a genetic distance matrix, calculates the minimum genetic distance among the different multilocus genotypes (i.e. threshold) and collapses the genotypes into distinct multilocus genotypes. For that, we used the function *cut-off* to define the threshold [see Supporting Information—Fig. S1] needed to cluster individuals into the same multilocus genotypes (hereafter clones) or distinct multilocus genotypes. Based on Euclidean genetic distance, we estimated the value was 10.30 to account for possible genetic variation among clones due to library preparation, sequencing error and somatic mutations. After the definition of the potential clones using the function *mgl.filter*, we estimated the geographic distance between them and between distinct multilocus genotypes, and the genotypic diversity (the number of genotypes divided by the number of ramets, *G*/*N*). We randomly sampled one clone individual to estimate the geographic distance between distinct multilocus genotypes. We used the Kolmogorov–Smirnov test to check for differences in the distance distributions between clone individuals and individuals with distinct multilocus genotypes.

### Fine-scale genetic structure and genetic diversity

To investigate the genetic divergence amongst sampled individuals of *D. filipedunculata*, we calculated the proportion of shared alleles using ‘adegenet’ R package (Jombart 2008), then plotted the results as a heatmap and a dendrogram showing individuals clustering using the ‘dartR’ R package (Gruber *et al.* 2018). We also tested for within-population genetic structure using the Bayesian analysis implemented in fastSTRUCTURE (Raj *et al.* 2014) with the full individuals dataset (49 clonal and non-clonal individuals) and the subset of 26 non-clonal individuals. The best *K* value was assessed from values between 1 and 10, as determined by their likelihood values using StructureSelector (Li and Liu 2018). Finally, we evaluated the fine-scale genetic structure (FSGS; i.e., the non-random spatial distribution of genotypes within populations) with both datasets using a spatial autocorrelation analysis as implemented in SPAGeDi (v. 1.5 software) (Hardy and Vekemans 2002). To do so, we set *Fij* as the kinship coefficient among individuals described in Loiselle *et al.* (1995) and defined 10 distance intervals. We permuted individuals’ spatial positions 1000 times to test for the significance of FSGS in each distance class.

We characterized the genetic diversity from the full dataset and the subset of non-clonal individuals using the number of alleles (*A*), observed (*H*_O_) and expected (*H*_E_) heterozygosities, and the inbreeding coefficient (FIS) with their confidence intervals based on 1000 bootstrap using the ‘diveRsity’ R packages (Keenan *et al.* 2013).

### Anatomical analysis

During fieldwork, we observed the underground structures of mature *D. filipedunculata* individuals (ca. 1 m in height, connected through small branches) and found that underground organs emitted new aerial branches. Thus, to identify these structures, we performed comparative anatomical analyses between the underground organs and the aerial branches. For this purpose, samples were collected from individuals (*n* = 3; with a distance of at least 5 m between each collection point) in the transition zone between the N1 plateau and the ombrophilous forest. Sampling of clonal structures followed Klimešová *et al.* (2019), in which adult plants without physical damage or signs of disease and nutritional deficiency were sampled. Sampled material was fixed in FAA 70 (37 % formaldehyde, glacial acetic acid, 70 % ethanol, 1:1:18 (v/v); Johansen 1940) and stored in 70 % ethanol. Subsequently, small fragments of aerial stems and underground organs were obtained, dehydrated in an n-butyl alcohol series and embedded in historesin (Leica Historesin Embedding Kit). Cross sections (5–7 µm thick) were obtained with a rotary microtome (Leica, RM 2255), stained with periodic acid–Schiff’s reagent (PAS) and toluidine blue (O’Brien *et al.* 1964; Feder and O’Brien 1968) and mounted on permanent slides using Entellan (Merck). The slides were analysed using a light microscope (Zeiss, Axio Scope A1) with an attached camera (Zeiss, AxioCam ICc 5) and AxioVision (version 4.8.3.0) software. Cross sections of the underground organs were sliced by hand and their general characterization was carried out under a stereomicroscope (Zeiss, SteREO Discovery V12) with an attached camera (Zeiss, AxioCam 712 colour) and ZEN 3.4 (blue edition) software. Histochemical tests were performed with Sudan III for lipids (Sass 1951) and ferric chloride for phenolic compounds (Johansen 1940).

### Sexual reproductive system

To verify whether *D. filipedunculata* reproduces sexually, we first analysed the floral morphology of individuals located in the transition zones between the ‘*canga*’ ecosystem and the ombrophilous forest (21 male and 10 female plants at the transition zone nearest to the N1 plateau, 2 female plants at the transition zone nearest to the N4 plateau, 1 female plant at the transition zone nearest to the N7 plateaus and 19 male plants at the transition zone nearest to the Lagoa da Mata; Fig. 1). Most individuals from N1 plateau are the same sample for genetic analysis; however, it is not possible to match them because they were sampled at different times and the individuals for genetic analysis were not marked. Since male and female plants are vegetatively similar, we first confirmed the sexuality of flowers from individuals that were flowering at the study sites from June to August 2022, observing the presence of androecium and gynoecium with a hand-magnifying lens. Additional individuals were analysed in June and July 2023 to increase sample size of female plants. To assess the existence of sexual dimorphism between floral types, we performed morphometric analyses of the perianth (hypanthium + corolla; length and width) and the gynoecium (length and width of the ovary, style length and stigma width). We collected 3 flowers from each of the 40 male plants and the 13 female plants, totalling 159 flowers analysed. The samples were fixed in FAA 70 and stored in 70 % ethanol. To perform the measurements, we took photographs on a stereomicroscope with a digital camera (the same equipment as that used in the anatomical analyses). We processed the images in ZEN 3.4 (blue edition) software.

We analysed the morphometric data using linear mixed models with a Gaussian distribution and the identity link function. Floral type (staminate or pistillate flowers) was considered a fixed factor, and the individual was considered a random factor to control pseudo-replicate effects. The best model was selected using likelihood ratio tests (Zuur *et al.* 2009), and the influence of the fixed factors on the response variable was analysed using post hoc Tukey tests. We performed all statistical analyses in R (R Core Team 2020) using the packages ‘lme4’ (Bates *et al.* 2015) and ‘emmeans’ (Lenth 2020).

To evaluate the functionality of flowers, we performed pollination tests (Richards 1997; Sage *et al.* 2005) on both floral types. For staminate flowers with developed pistillodes, we used the 40 male plants and marked 6 flower per individual, 1 for each test and performed the following treatments [see Supporting Information—Table S1]: (a) spontaneous self-pollination, in which preanthesis buds were isolated with fabric bags, preventing pollination by external agents; (b) manual self-pollination, in which preanthesis buds were bagged and, during anthesis, pollinated with their own pollen; (c) geitonogamy, in which preanthesis buds were emasculated and bagged, and then flowers at anthesis were pollinated with pollen from flowers of the same individual; (d) cross-pollination, in which preanthesis buds were emasculated and bagged, and at the time of anthesis, the flowers were pollinated with pollen from flowers of different individuals; (e) pollen supplementation in which we performed manual outcross pollen addition without bagging the flowers and (f) control (open pollination), in which preanthesis buds were marked and during anthesis were exposed to local environmental conditions. We performed all crosses on the first day of anthesis with fresh pollen. We carried out manual pollinations (treatments b–e) with the help of wooden toothpicks, replacing them in each flower to avoid contamination. For treatments (d) and (e), we used individuals as pollen donors, whose preanthesis flowers were bagged to avoid pollen loss. We collected pollen from individuals at least 10 m away from the individuals that would be pollen recipients. According to distance and flower availability, different sets of individuals were used as pollen donors.

For pistillate flowers, we used 13 plants and marked 4 flowers per individual 1 for each test, and performed the following treatments: (a) apomixis, in which preanthesis buds were isolated with fabric bags, preventing pollination by external factors; (b) cross-pollination, in which preanthesis buds were bagged and at anthesis, the flowers were pollinated using pollen from individuals with staminate flowers; (c) pollen supplementation, similar to the corresponding condition described above and (d) control, similar to the corresponding condition described above.

**Figure 2.**
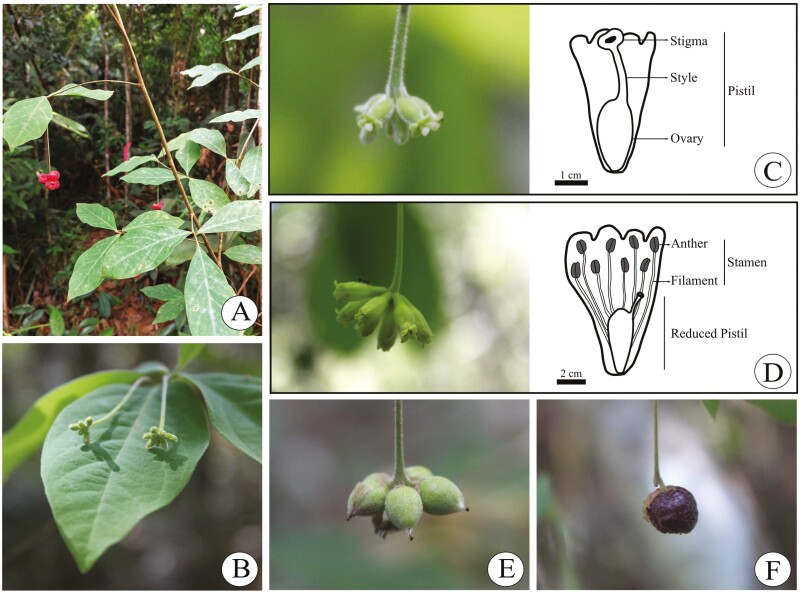
Characterization of *D. filipedunculata*. (A) Habit, (B) nflorescences with buds, (C) pistillate flowers, (D) staminate flowers, (E) immature fruits and (F) ripe fruits of *D. filipedunculata*.

We followed all the reproductive phases of the individuals in the population, such as floral buds, flowers at anthesis and immature and mature fruits (Fig. 2). In general, we verified the possible formation of fruits (berry-like, containing one seed) 1 month after we carried out the treatments in the field. Differences in fruit formation among treatments were compared with chi-squared tests, and *P* values ≤ 0.05 were considered to indicate a significant difference.

## Results

### Identification of clonemates using high-throughput sequencing

**Figure 3.**
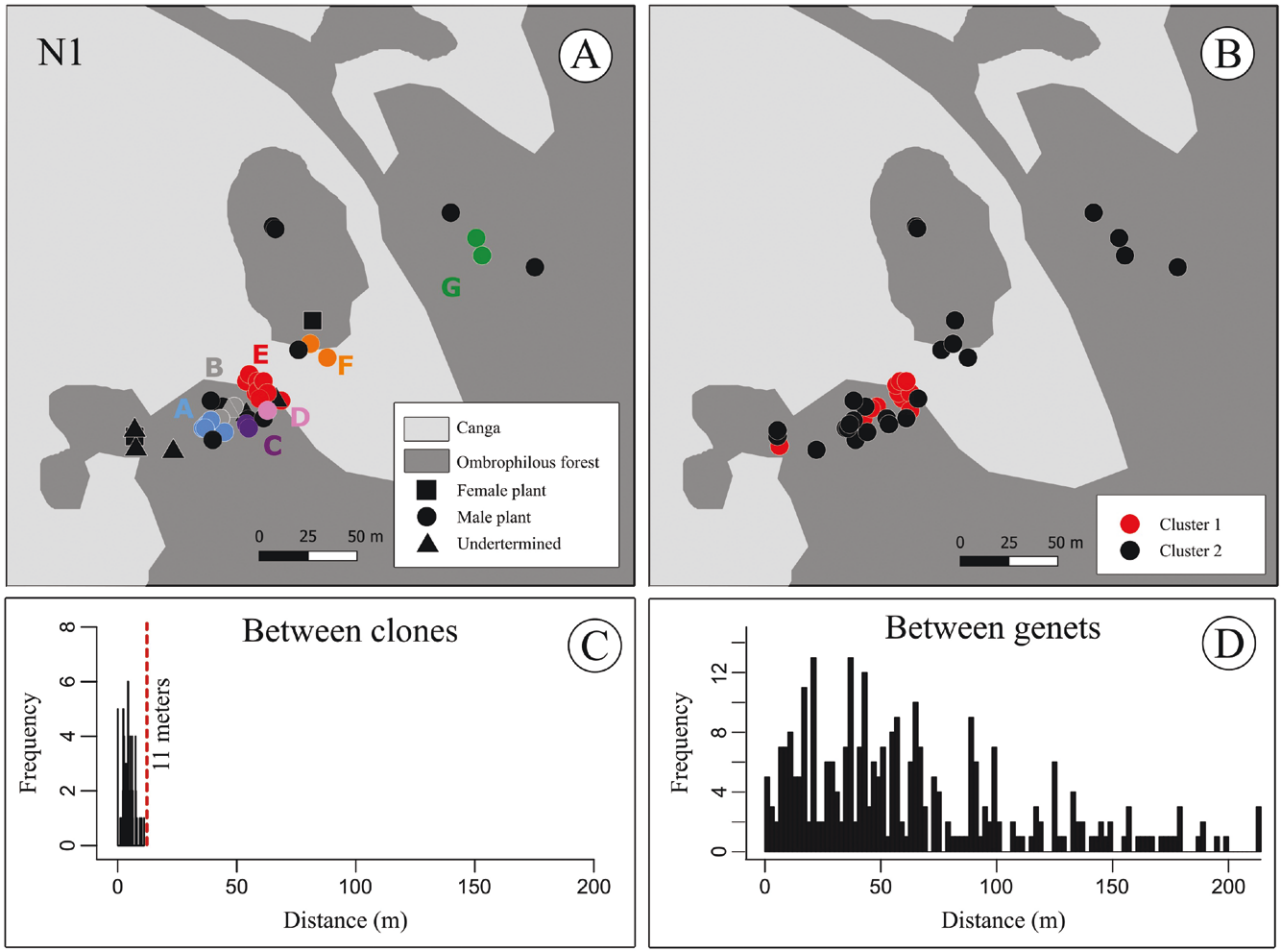
Fine-scale distribution and geographic distance among sampled individuals of *D. filipedunculata* in the transition zone between N1 canga plateau and the ombrophilous forest, FLONA de Carajás, Eastern Amazon, Brazil. (A) Spatial distribution of clonal and non-clonal individuals. Distinct letters (A–G) and colours correspond to individuals identified as clones based on genetic analysis using the dataset of 1122 SNPs. (B) Geographic distance between *D. filipedunculata* individuals with the same multilocus genotypes (clonal), (C) and between individuals belonging to distinct multilocus genotypes (non-clonal).

We retained 1122 SNPs across 49 individuals of *D. filipedunculata* for clonal identification. The number of distinct multilocus genotypes found across the 49 individuals was 26, and the G/N ratio was 0.53 (Supporting Information—Table S2, Fig. 3A). The number of individuals by multilocus genotypes is *A* = 5, *B* = 3, *C* = 2, *D* = 2, *E* = 12, *G* = 3 and *F* = 3. The geographic distance between clonal individuals ranged from less than a metre to 11 m, with a median of 4 m (Fig. 3C). In contrast, the geographic distance between distinct multilocus genotypes ranged from 0 to 213 m, with a median of 51 m (Fig. 3D). These distance distributions were significantly different (Kolmogorov–Smirnov test, *D* = 0.85, *P* < 2.2e-16). Interestingly, we found one case of identical multilocus genotypes (clones) presenting female and male individuals [Supporting Information—Table S2].

### Fine-scale genetic structure and genetic diversity

**Figure 4.**
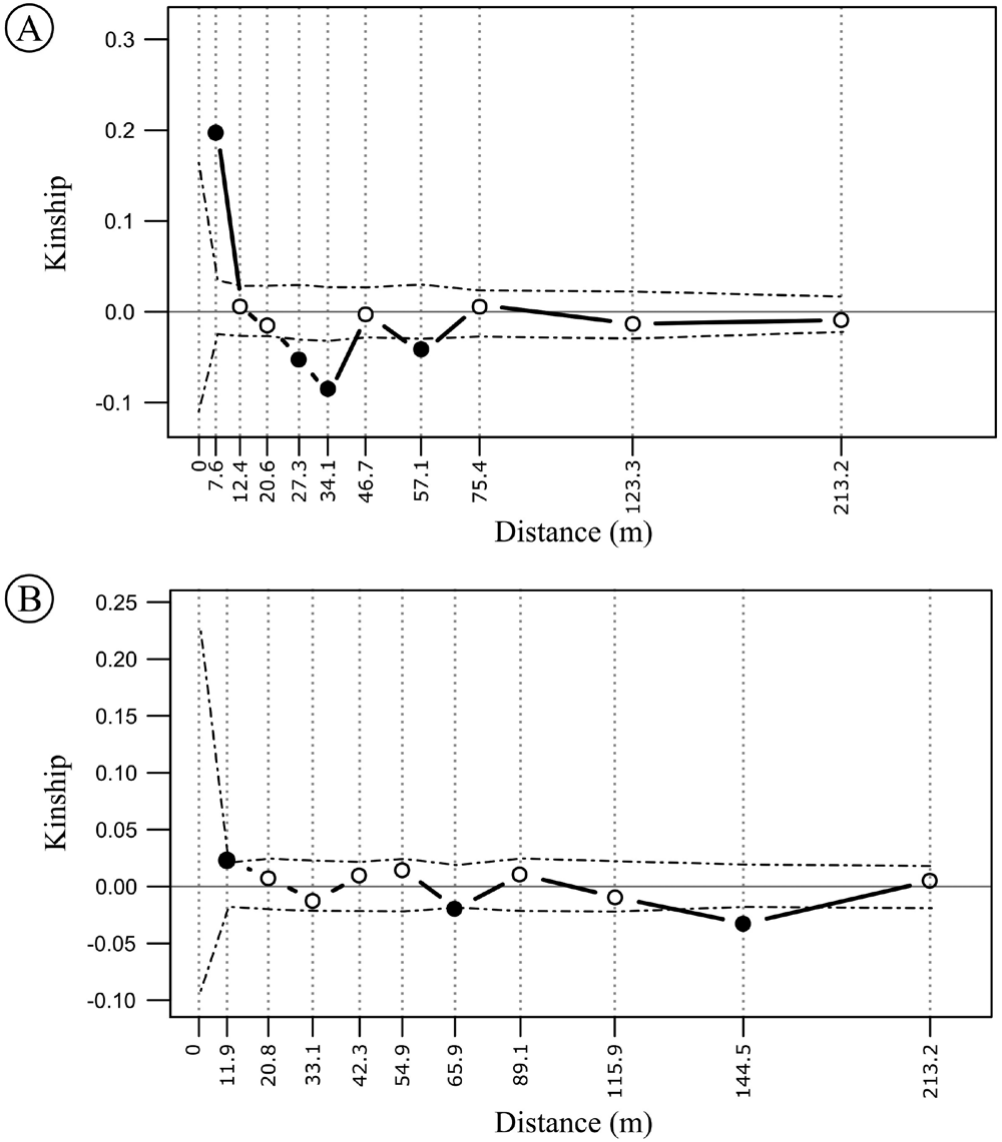
FSGS of *D. filipedunculata* in the transition zone between N1 *canga* plateau and the ombrophilous forest, FLONA de Carajás, eastern Amazon, Brazil. (A) Correlogram for the spatial autocorrelation analyses for 10 distance intervals using kinship index calculated between all pairs of clonal and non-clonal individuals. (B) and between the subset of all pairs of non-clonal individuals. Kinship above or below permuted 95 % confidence intervals (dashed lines) are represented by filled symbols.

While non-clonal individuals share 80.03–92.69 % of the sampled alleles, distinct individuals identified as belonging to the same genet (clones) have 97.77–99.95 % of identity [Supporting Information—Fig. S2]. The analysis performed in fastSTRUCTURE from the full dataset of individuals recovered two distinct genetic clusters mixed in the geographical space (Fig. 3B, Supporting Information—Fig. S3A). The individuals with the greatest genetic mixture were those with distinct multilocus genotypes in the population (Supporting Information—Fig. S3, individuals from 18 to 32). Such substructure within the sampled area was not corroborated by the analysis performed with the dataset of non-clonal individuals [see Supporting Information—Fig. S3B]. The spatial autocorrelation analysis performed from the full dataset showed a pattern of sharp decrease of kinship with increasing geographical distance, up to the sixth distance class (Fig. 4A). When clonal individuals are not included in the analysis, such trend disappears (i.e. kinship is not different from the expected by chance for any but three distance class, Fig. 4B). We found no evidence of inbreeding (FIS = −0.027 [−0.064 to −0.003] or −0.0069 [−0.0419 to 0.0122], including or not the clonal individuals, respectively) and a moderate level of genetic diversity (*H*_E_ = 0.180 and 0.190, including or not clonal individuals, respectively) in *D. filipedunculata* [see Supporting Information—Table S3].

### Comparison of root and stem anatomy

**Figure 5.**
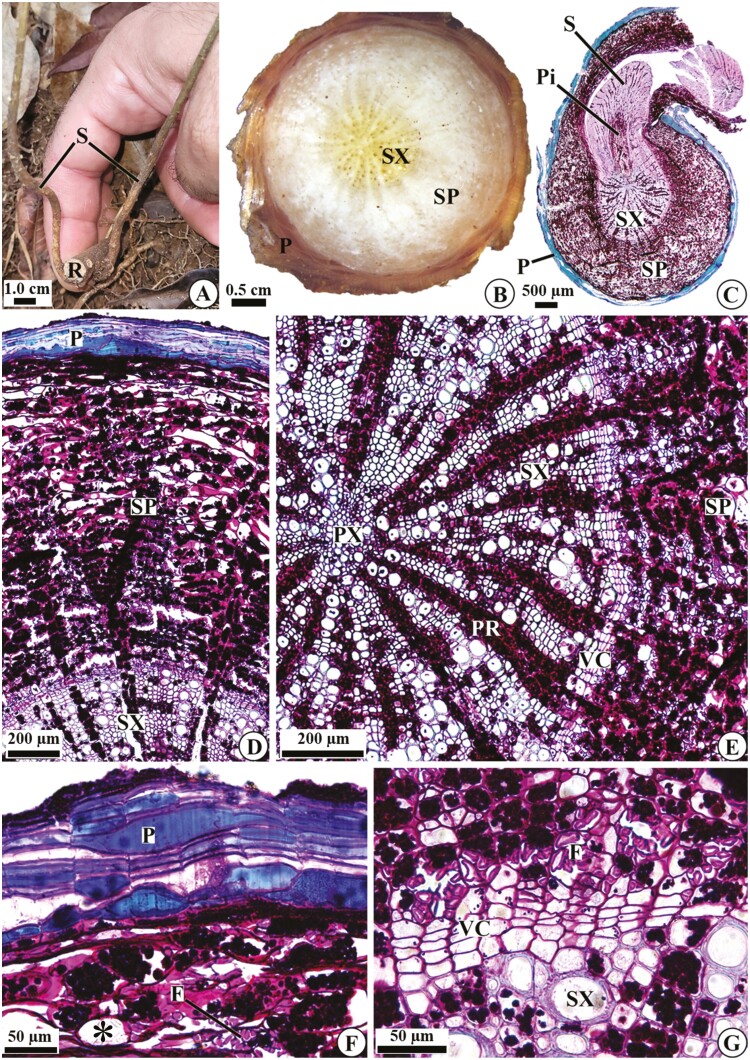
Plant architecture (A) and root anatomy (B–G) of *D. filipedunculata* shown in cross sections. (A) Vegetative structure; note the two sprouts originating from one root. (B–C) General aspects: in C, a sprout is connected to the vascular system. (D) Details of the periderm, secondary phloem and secondary xylem. (E) Details of the secondary phloem, vascular cambium and secondary and primary xylem. (F) Details of the periderm and part of the secondary phloem. (G) Details of the vascular cambium and part of the secondary phloem and secondary xylem. F, fibres; P, periderm; Pi, pith; PR, parenchymatous ray; PX, primary xylem; R, root; S, stem; SP, secondary phloem; SX, secondary xylem; VC, vascular cambium; *, idioblast.

**Figure 6.**
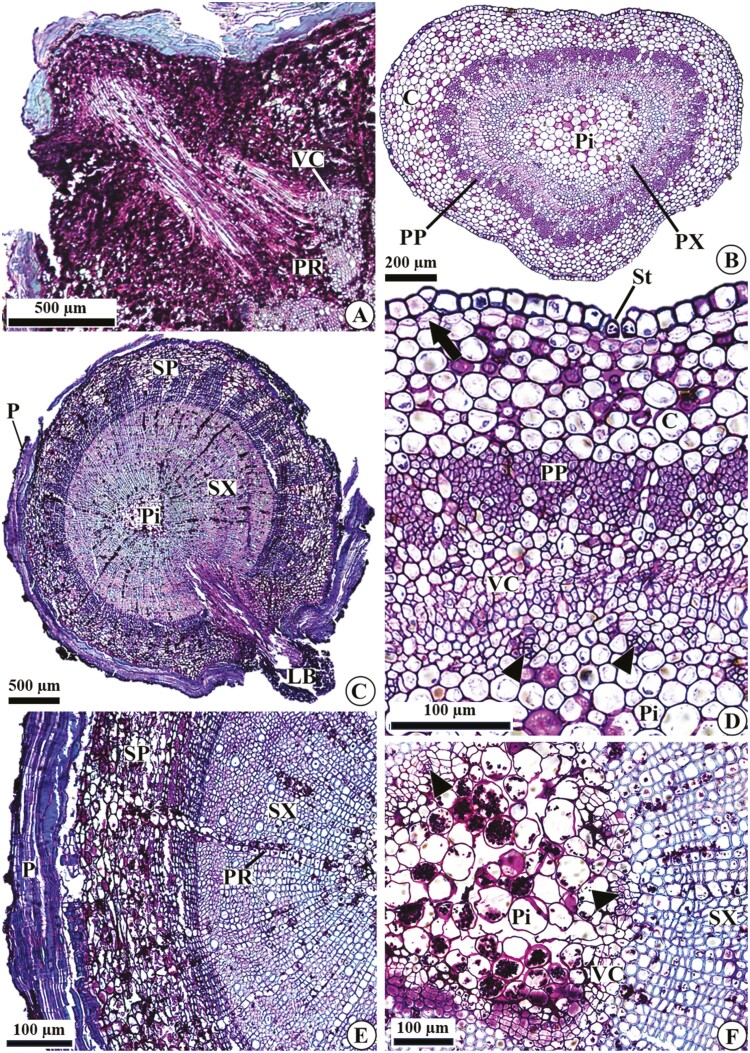
Stem anatomy of *D. filipedunculata* in longitudinal (A) and cross (B–F) sections. (A) Part of the sprout connected to the root, associated with a wide parenchymatous ray. (B–C) General aspects in the apical and basal regions. (D) Details of the primary structure, with the establishment of phellogen below the epidermis. (E) Details of the secondary structure, with periderm, secondary phloem and secondary xylem. (F) Details of the pith in a stem with secondary growth. Arrow, phellogen; arrowheads, primary xylem; C, cortex; P, periderm; Pi, pith; PP, primary phloem; PR, parenchymatous ray; PX, primary xylem; SP, secondary phloem; St, stomata; SX, secondary xylem; VC, vascular cambium.

We found that the clonal propagation of *D. filipedunculata* occurs through the formation of new sprouts (aerial branches) from underground organs (Fig. 5A and C). Our observations revealed differences in the structure of the aerial branches and underground system, which allowed the characterization of underground organs as roots (absence of a pith and centripetal maturation of the primary xylem). Roots in the secondary structure consist of periderm and secondary phloem and xylem (Fig. 5B–D). The outer layers of the periderm (suber) have a loose arrangement, with evident lacunae and cells containing phenolic compounds and suberized walls (Fig. 5D and F). Secondary phloem occurs between the vascular cambium and periderm, characterized mainly by parenchymatous cells containing starch grains and fibres (Fig. 5D–G); idioblasts containing crystals may occur (Fig. 5F—asterisk). The vascular cambium has three to four layers of flat, thin-walled cells (Fig. 5E and G). Regarding the xylem, the secondary structure is quite evident compared to the primary structure, presenting conducting cells and many parenchymatous rays, with cells storing starch grains (Fig. 5E and G). The aerial branches are connected to the vascular cambium and secondary xylem (Fig. 5C); these sprouts develop in regions where the parenchyma rays of the secondary xylem are wider (Fig. 6A), connecting the reserve tissues of the root and stem.

The stem anatomy differs from that of the root mainly due to the presence of a parenchymatous pith (Figs 5C and 6B and C). In the apical portion, the stem exhibits a primary structure or initiates secondary growth (Fig. 6B and D); a single-layered epidermis and a parenchymatous cortex are still present. In the basal portion, the stem is composed of periderm, vascular cambium and secondary phloem and xylem (Fig. 6C and E), with characteristics similar to those of the roots; the only difference is the narrower parenchymatous rays in the secondary xylem of the stems (Fig. 6E). In stems with secondary growth, the pith starts to store starch grains, and some of its cells rupture, forming intercellular spaces (Fig. 6F).

### Sexual reproductive system

**Figure 7.**
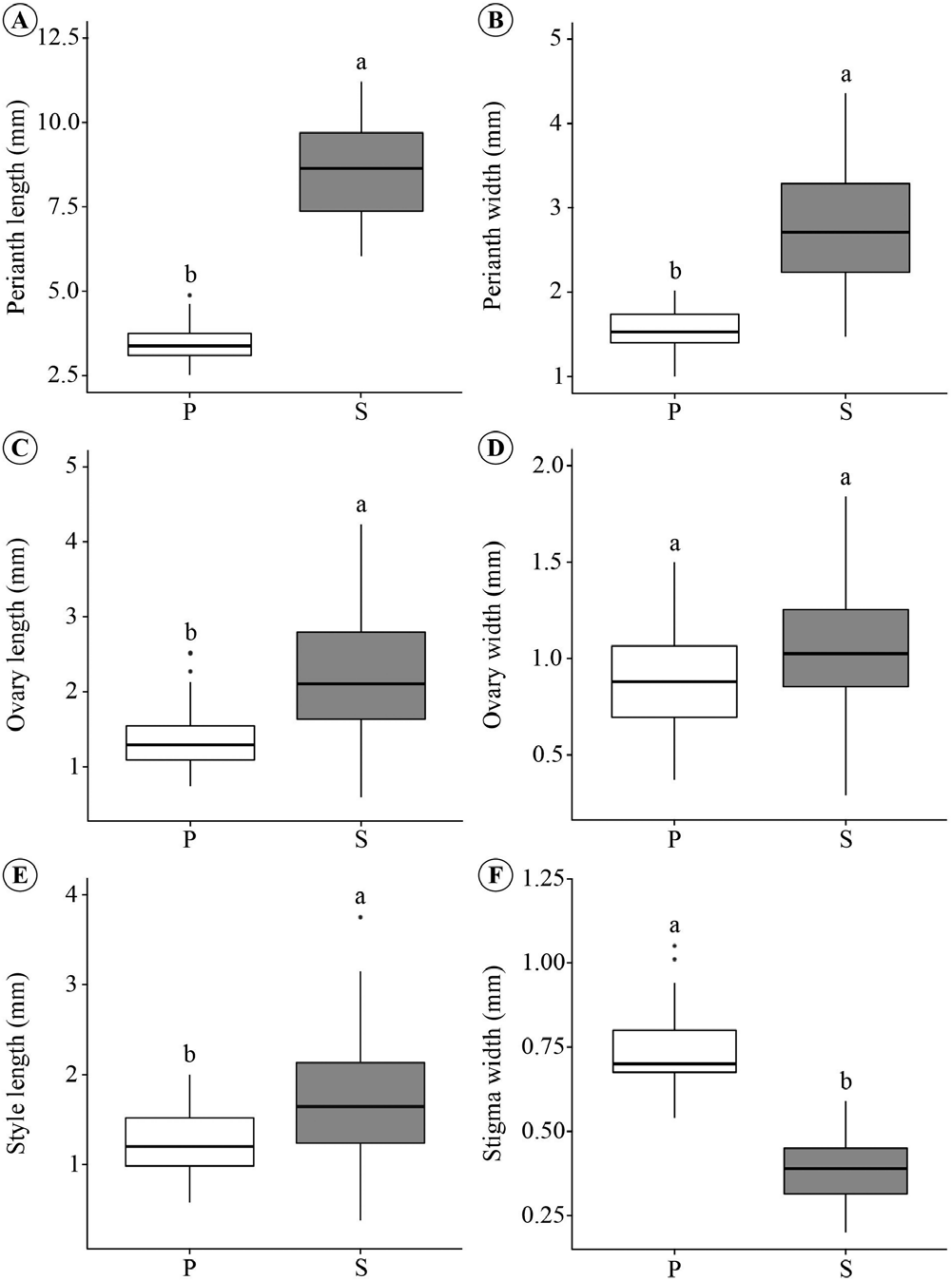
Floral morphometry of *D. filipedunculata*. (A) Perianth length. (B) Perianth width. (C) Ovary length. (D) Ovary width. (E) Style length. (F) Stigma width. Averages followed by the same lowercase letter indicate floral traits that did not differ significantly (at *P* ≤ 0.05) between floral types according to Tukey’s post hoc test. P, pistillate flower; S, staminate flower with developed pistillode.

The floral types of *D. filipedunculata* have morphometric differences [see Supporting Information—Table S4]. The perianth of staminate flowers with developed pistillodes is larger than that of pistillate flowers (Fig. 7A and B). The gynoecium also differs between floral types, with the length of the ovary and style greater in staminate flowers with developed pistillodes (Fig. 7C and E), while the width of the stigma was greater in pistillate flowers (Fig. 7F). Ovary width did not differ between floral types (Fig. 7D). Pistillate flowers do not have staminodes. The androecium of staminate flowers with developed pistillodes consists of 8 (6–12) stamens that have fillets fused with the perianth; the anthers are arranged at two heights (see Fig. 2).

*Daphnopsis filipedunculata* staminate flowers with developed pistillodes did not produce fruits in any of the applied treatments [see Supporting Information—Table S1]. Pistillate flowers did not produce fruit by apomixis or in the pollen supplementation treatment, and fruitification in the other treatments was low; open pollination led to the highest reproductive success (23.1 %) [see Supporting Information—Table S1]. However, there were no significant differences in fruit formation among the treatments in pistillate flowers (*X*^2^ = 4.25; *P* = 0.23).

## Discussion

Sexual reproduction is vital to ensure variation within species and promote adaptation to novel environmental conditions, while asexual reproduction, a common feature of plants, can help some species escape failure of sexual reproduction and potentially preserve well-adapted phenotypes in environmentally stable sites (Silvertown 2008; Niklas and Cobb 2017; Tandon *et al.* 2020). Here, we showed that clonal propagation plays an important role in the *D. filipedunculata* population. Specifically, we demonstrated that this species presents female and functional male phenotypes, indicating that *D. filipedunculata* is an obligate xenogamous species but has low reproductive success. We also showed that clonal propagation is the main mode of reproduction in *D. filipedunculata* and that roots are the organ responsible for clonal growth. Finally, our results indicated that *D. filipedunculata* presents an intermediate phalanx–guerrilla clonal architecture, as the distance between ramets from the same genet was usually short, but the spatial distribution of individuals showed a mixture of ramets from different genotypes. Our findings have important implications for the conservation of this endangered, highly endemic species and throughout the discussion, we have suggested future studies to carry out with this species.

The flowers of *D. filipedunculata* exhibit dimorphisms in primary (pistil) and secondary (perianth) sexual characteristics (Sakai and Weller 1999). Dimorphic species generally exhibit staminate flowers that have a larger perianth than that of pistillate flowers (Freeman *et al.* 1997; Eckhart 1999), as observed here. This difference may be associated with the attraction of pollinators, considering that the fitness of staminate flowers depends more strongly on mating success (Bell and Hamilton 1985; Eckhart 1999). In relation to the gynoecium, the size of the pistillode of staminate flowers may be similar to or larger than that of the pistil of pistillate flowers, a common characteristic in the subgenus *Neivira* (Nevling Jr. 1959). This morphological similarity could be mistakenly interpreted as evidence of gynodioecy (hermaphrodite and female individuals), a sexual expression present in other genera of Thymelaeaceae (Medrano *et al.* 2005; Beaumont *et al.* 2006; Merrett 2007; Shibata and Kudo 2017). However, the loss of female function in the staminate flower occurred due to the atrophy of the pistillode stigma, which we confirmed by the absence of fruiting in all pollination tests carried out in this floral type. Thus, *D. filipedunculata*, as well as the other species of the subgenus *Neivira* that have a well-developed pistillode (Nevling Jr. 1959; Barringer and Pruski 2005), is cryptic dioecious (*sensu*Mayer and Charlesworth 1991), differing from the other species of the genus that have reduced pistillodes (Nevling Jr. 1959, 1963; Nevling and Barringer 1986; Breedlove and de la Luz 1989) and are morphologically dioecious (*sensu*Mayer and Charlesworth 1992).

The existence of cryptic dioecy in *D. filipedunculata* plays an important role in inbreeding avoidance (Mayer and Charlesworth 1991), as confirmed by the inbreeding coefficient equal to zero and moderate genetic diversity calculated from our genomic dataset. These values of genetic diversity are similar to those found in other obligate xenogamous endemic plant (*Ipomoea cavalcantei*, Lanes *et al.* 2018), but smaller than those observed in endemic species with wider distributions in this region (*Brasilianthus carajensis* and *Monogereion carajensis*, Carvalho *et al.* 2019). According to Mayer and Charlesworth (1991), non-functional sexual parts may be found in flowers because insufficient evolutionary time has passed to lead to their suppression, indicating that these populations are transitioning to morphological dioecy. Dioecy in *D. filipedunculata* must have originated with a hermaphrodite ancestor through an intermediate gynodioecious stage, a route also proposed for other species in the family (Burrows 1960; Mayer 1990; Alonso and Herrera 2011; Shibata *et al.* 2018). The fact that staminate flowers with developed pistillodes morphologically resemble a bisexual flower reinforces this idea. Dioecious species that evolved by this route should neither have individuals who change sex in response to increasing age or heterogeneous environments nor should there be spatial segregation between male and female plants (Freeman *et al.* 1997). Nevertheless, genetic analyses revealed one case of identical multilocus genotypes presenting female and male individuals; thus, further investigation is needed to confirm that *D. filipedunculata* is sexually labile. Sex change in individuals is expected to be a random, nonadaptive and rare event in dioecious species that evolved via gynodioecy (Freeman *et al.* 1997), with this change better defined as sexual inconstancy (*sensu*Lloyd and Bawa 1984).

We observed a male-biased ramet sex ratio in *D. filipedunculata*, corroborating the findings of Watanabe *et al.* (2018), who reported a lower frequency of female individuals than male individuals. Most of the clones detected in our study were male plants, but these data must be interpreted with caution, as we found few female plants in the area (personal observation). Moreover, the number of individuals varies among clones and may be related to age, fitness or habitat, factors that were not tested in our study. Dioecious species typically present male-biased populations (e.g. Queenborough *et al.* 2013; Khanduri *et al.* 2019), although female-biased populations can occur (see Wang *et al.* 2013). The prevalence of male-biased populations is associated with the higher reproductive costs involved in the production of fruit and seeds by female plants, which are often associated with reduced survival rates, thus affecting population sex ratios (Obeso 2002; Barrett and Hough 2013; Lin *et al.* 2015). Differences in investment in sexual reproduction can also explain the rates of clonal propagation between sexes (Field *et al.* 2013). Male plants invest less in reproduction and thus likely have more resources for clonal growth, favouring a male-biased ramet sex ratio (Sinclair *et al.* 2012; Field *et al.* 2013). Interestingly, in a gynodioecious *Gnidia* species, Beaumont *et al.* (2006) observed that female plants invested 7.3 % more energy in reproduction than hermaphrodite plants. In contrast to observations in *Gnidia*, *D. filipedunculata* staminate flowers with developed pistillodes (morphologically hermaphrodite) did not produce fruits, which can increase the availability of resources for male plants, increasing their fertility, growth or survival (Mayer and Charlesworth 1991). Further investigations are needed to identify whether reproductive costs are related to the higher frequency of clonal male ramets in *D. filipedunculata*.

The absence of fruiting by apomixis in *D. filipedunculata* indicates that this dioecious species is dependent on pollination vectors for fruit formation to occur, in contrast to other species in the family, which are hermaphrodite and apomictic (Williams 2004; Graves 2008). Moreover, the lack of fine-spatial genetic structure among non-clonal individuals likely indicates that dispersal by pollen or seeds is not limited by distance in this species. Thymelaeaceae species are visited by insects from different orders, such as Coleoptera, Diptera, Hymenoptera, Hemiptera, Lepidoptera and Thysanoptera (de la Bandera and Traveset 2006; Roccotiello *et al.* 2009; Rodríguez-Pérez and Traveset 2011; Sakata and Nakahama 2018), and are possibly an ecologically and functionally generalist pollination system (*sensu*Ollerton *et al.* 2007); however, there are also some cases of pollination by moths (Makholela and Manning 2006; Okamoto *et al.* 2008; Chen *et al.* 2016). Nevertheless, fruit set tends to be <50 % (Williams 2004; Roccotiello *et al.* 2009; Rodríguez-Pérez and Traveset 2011; Sakata and Nakahama 2018; Shibata *et al.* 2021), similar to the rates observed in this study, and there may be annual variation (Schulz *et al.* 2004; Roccotiello *et al.* 2012). Pollen limitation due to the lack of compatible mates and pollinator behaviour have been identified as key factors explaining the low fruitification among dioecious and self-incompatible species (e.g. Wilcock and Neiland 2002; Faria *et al.* 2006; Vallejo-Marín *et al.* 2010; Barros *et al.* 2013; Ferreira *et al.* 2022). However, pollen supplementation did not favour fruit set in *D. filipedunculata* and we found markedly higher genotypic diversity (*G*/*N* = 0.53 for all individuals and *G*/*N* = 0.44 after removing female and undetermined ramets). As the studied population is male-biased, the high *G*/*N* ratio could reflect a high diversity of males for pollen donation. Thus, such results are the opposite of those expected in dioecious species. Other factors may be related to the low fruiting rate, such as plant size, light availability (Schulz *et al.* 2004), the short duration of pollen grain viability or stigmatic receptivity (Roccotiello *et al.* 2009).

Asexual reproduction in angiosperms can be achieved via clonal propagation and apomixis (Barrett 2015). Since we did not observe fruit formation by apomixis, we concluded that clonality is the main form of asexual reproduction in *D. filipedunculata*. Patterns of clonal propagation, such as clonal architectures and growth forms, are expected to affect mating systems and sexual reproductive success because they are the main factors responsible for the spatial distribution of ramets (Vallejo-Marín *et al.* 2010; Barrett 2015). Here, we found that roots are the main organ responsible for vegetative propagation in *D. filipedunculata* and that the distance between ramets from the same genet is usually short resulting in fine-scale genetic structure within the N1 transition area. Nevertheless, the spatial distribution of individuals showed a mixture of ramets from different genotypes. These observations suggest that *D. filipedunculata* presents an intermediate phalanx–guerrilla clonal architecture (Vallejo-Marín *et al.* 2010). Our results also show two genetic clusters when clonal and non-clonal individuals are included in genetic structure analysis, showing that although there is a mixture of ramets from different genotypes, some clonal groups are genetically very similar. This pattern disappears when only non-clonal individuals are analysed, suggesting that the inclusion of individuals with the same genotypes forces the formation of more genetic groups.

Clonal propagation, especially through underground organs, represents a general strategy of resource storage (Klimešová *et al.* 2018; Franklin *et al.* 2021), which may include starch, as seen in *D. filipedunculata*. Interestingly, root sprouting is frequently associated with environmental disturbances such as windthrows and fires (Klimešová *et al.* 2017). We found that the gemmiferous roots of *D. filipedunculata* exhibit regular secondary growth (establishment of a single vascular cambium), with the cambium forming a large proportion of the parenchyma tissue storing starch grains; thus, these roots are characterized as tuberous (Appezzato da Glória 2015). Gemmiferous roots can develop two types of buds, additional and reparative, with the former associated with undisturbed environments and the latter associated with physical damage (i.e. injuries and fire). While the reparative buds have an exogenous origin from external tissues of the root, the additional buds have an endogenous origin and may develop from internal tissues such as the secondary phloem and vascular cambium, leaving traces contiguous with the centre of the root (Appezzato da Glória 2015). Although we did not carry out an ontogenetic study to characterize the root buds, we observed a continuity of vascular tissues between roots and new ramets, which suggests that they are additional buds. This indicates that in *D. filipedunculata*, clonal propagation occurs naturally, that is, not mediated by any root damage.

Besides the importance of disturbance events (Klimešová *et al.* 2017), the natural formation of bud by roots has been previously reported (e.g. Klimešová and Martínková 2004), and environmental heterogeneity has been pointed as a key trigger of root sprouting (see Klimešová *et al.* 2017). *Daphnopsis filipedunculata* occurs in the transition zone between ironstone outcrops and ombrophilous forests (Watanabe *et al.* 2018), which is a more heterogeneous habitat than those from mature forests where the species was recently found (unpubl. data). Thus, resource sharing (evidenced by starch grains stored in the stems and roots of ramets of a single genet) represents an efficient strategy for clonal survival, avoiding or minimizing intraindividual competition, especially in the understory, where resources such as light, water and soil nutrients are heterogeneously distributed throughout the habitat (Lei 2010; Guo *et al.* 2011; Duchoslavová and Herben 2020). Compared to the stems, the roots are deeper, protecting the buds that form the new ramets (Klimešová *et al.* 2019). Yet, buds can originate from different portions of the root system with no morphological limitations (i.e. by node positions as in rhizomatous and stoloniferous species), and new shoots possess its own root system with no time limitations such as those from rhizomatous and stoloniferous plants, where the formation of adventitious root system is often delayed (Klimešová and Klimeš 2008; Martínková *et al.* 2018). The costs and constraints in root bud formation and resource sharing in this manner will need further investigation, but the functional integration observed between taller ramets and new sprouts might be a key step in the success of clonality in *D. filipedunculata*, as it prolongs the life of clonal individuals and reduces the risk of mortality of these new ramets in the area (Lei 2010; Duchoslavová and Herben 2020; Shi *et al.* 2021).

We found low fruit production that pollen supplementation did not increase and a moderate proportion of clones in the studied population. The expansion of clonality can impact seed production by limiting the resources allocated to flowering (Van Drunen and Dorken 2012) and by constraining mating opportunities in self-incompatible species (Honnay and Jacquemyn 2008; Hu *et al.* 2017) or in species with sexual polymorphism (Widén and Widén 1990; Wang *et al.* 2005; Barrett 2015), as observed in our study. Despite the impact of clonality on fruit production, clonal propagation has been identified as a driver of population abundance and persistence in species with low fruit and seed sets (Faria *et al.* 2006). Such trade-offs between low seed production and clonal propagation as well as compensatory responses have been reported for self-incompatible and dioecious species (Herben *et al.* 2012; Van Drunen and Dorken 2012; Barrett 2015; Hu *et al.* 2017). Environmental factors seem to drive the balance between sexual and asexual reproduction (Honnay and Bossuyt 2005; Binks *et al.* 2015; Hewitt 2020); thus, future studies of *D. filipedunculata* should investigate whether the balance between sexual reproduction and clonal propagation differs throughout its distribution range, conducting detailed analyses of the costs associated with these types of reproduction (e.g. Lei 2010; Franklin *et al.* 2021).

### Conservation implications

Our results have important implications for the management and conservation of *D. filipedunculata*, a highly restricted endemic species. First, the extensive clonal growth indicates that population size estimates for the species should consider that many apparently distinguishable individuals may actually represent a single genet. Second, the finding that individuals within 11 m from each other are more likely to be clones implies that one should avoid sampling more than one plant within such a radius threshold for *ex situ* conservation, such as the establishment of genetically representative *ex situ* collections, seed banks or translocated populations (Bragg *et al.* 2020). It is also important to consider, for *ex situ* collection, sampling plants during their reproductive period to ensure collection from both sexes. Our data also provide the first insights into the relative importance of sexual reproduction and clonal propagation in *D. filipedunculata*, an important aspect that should inform the management, conservation and restoration practices implemented for threatened species (Sydes and Peakall 1998; Cascante-Marín *et al.* 2020). Nevertheless, further research evaluating the extent of clonality in other habitats where the species occurs naturally, as well as the impact of clonality versus sexual reproduction on population growth rates, is needed since such patterns may vary over time and may be dependent on the environmental context (e.g. Dorken and Eckert 2001; Xie *et al.* 2001; Kimura *et al.* 2013). Notably, prolonged and nearly exclusive clonal growth could ultimately lead to local sexual extinction in *D. filipedunculata* with significant consequences for population viability (see Honnay and Bossuyt 2005). The lack of genetic variation due to increased clonality could also constrain long-term adaptation to environmental changes (Lynch 1995; Barrett and Schluter 2008) and needs to be extensively investigated across the entire distribution range of the species.

## Supporting Information

The following additional information is available in the online version of this article –

**Table S1**. Pollination test results of floral types of *Daphnopsis filipedunculata* in the FLONA de Carajás, Brazil.

**Table S2.** Multilocus genotypes that occurred in more than one individual of *Daphnopsis filipedunculata* in the FLONA de Carajás, Brazil.

**Table S3:** Characterization of the genetic diversity of *Daphnopsis filipedunculata* based on 1122 SNPs sampled in 49 clonal and non-clonal individuals (full dataset) and a subset of 26 non-clonal individuals.

**Table S4.** Summaries of the linear mixed models used to analyse floral traits of *Daphnopsis filipedunculata* in the FLONA de Carajás, Brazil.

**Figure S1.** Threshold of Euclidean genetic distance (vertical line) among individuals of *Daphnopsis filipedunculata* in the FLONA de Carajás, Brazil.

**Figure S2**. Heatmap (A) and histogram (B) showing the proportion of shared alleles amongst 49 individuals of *Daphnopsis filipedunculata*.

**Figure. S3.** Barplot showing fastSTRUCTURE results for the full dataset of 49 clonal and non-clonal individuals and a subset of 26 non-clonal individuals of *Daphnopsis filipedunculata.*

plae048_suppl_Supplementary_Materials

## Data Availability

Floral traits and mating system data, and geographic coordinates in decimal degrees and the genotypes in Variant Call Format are provided in figshare: 10.6084/m9.figshare.26836693. Raw reads have been deposited in the NCBI SRA database (BioProject ID: PRJNA1153566).
