# Supplementary material for: Mating system, morphological and genetic evidence endorse clonality as an essential reproductive mode in Daphnopsis filipedunculata (Thymelaeaceae), a dioecious and endemic species from the Amazon
Source: AoB Plants. 2024 Sep 10;16(5):plae048. doi: 10.1093/aobpla/plae048 (PMC11459258; doi:10.1093/aobpla/plae048)
Supplement: plae048_suppl_Supplementary_Materials [file plae048_suppl_supplementary_materials.docx]

**Supplementary Material**

**Mating system, morphological, and genetic evidence endorse clonality as an essential reproductive mode in *Daphnopsis filipedunculata* (Thymelaeaceae), a dioecious and endemic species from the Amazon**

**Table S1**: Pollination test results of floral types of *Daphnopsis filipedunculata* in the FLONA de Carajás, Brazil. n.a., not applicable; RS, reproductive success.

| Treatments | Staminate flower with developed pistillode | | | Pistillate flower | | |
| --- | --- | --- | --- | --- | --- | --- |
|  | Flower | Fruit | RS (%) | Flower | Fruit | RS (%) |
| Spontaneous self-pollination | 40 | 0 | 0 | n.a. | n.a. | n.a. |
| Apomixis | n.a. | n.a. | n.a. | 13 | 0 | 0 |
| Manual self-pollination | 36 | 0 | 0 | n.a. | n.a. | n.a. |
| Geitonogamy | 36 | 0 | 0 | n.a. | n.a. | n.a. |
| Cross-pollination | 36 | 0 | 0 | 11 | 1 | 9.1 |
| Pollen supplementation | 40 | 0 | 0 | 8 | 0 | 0 |
| Open pollination | 40 | 0 | 0 | 13 | 3 | 23.1 |

**Table S2:** Multilocus genotypes that occurred in more than one individual of *Daphnopsis filipedunculata* in the FLONA de Carajás, Brazil. The columns indicate the number of individuals of the same genotype (clones) that were identified as female plant, male plant, or undetermined. The color column is according to Figure 3.

| **Multilocus genotype** | **Color**  **(Fig. 3)** | **Female** | **Male** | **Undetermined** |
| --- | --- | --- | --- | --- |
| A | blue | 0 | 5 | 0 |
| B | gray | 0 | 2 | 1 |
| C | purple | 0 | 2 | 0 |
| D | pink | 0 | 2 | 0 |
| E | red | 0 | 11 | 1 |
| G | green | 1 | 2 | 0 |
| F | orange | 0 | 3 | 0 |

**Table S3:** Characterization of the genetic diversity of *Daphnopsis filipedunculata* based on 1,122 SNPs sampled in 49 clonal and non-clonal individuals (full dataset) and a subset of 26 non-clonal individuals. N = number of individuals, A = number of alleles, H_O_ = observed heterozygosity, H_E_ = expected heterozygosity and FIS = coefficient of inbreeding (with confidence intervals).

| Dataset | N | A | H_O_ | H_E_ | FIS |
| --- | --- | --- | --- | --- | --- |
| Full dataset | 49 | 1998 | 0.190 | 0.180 | -0.027 (-0.064 to -0.003) |
| Subset non-clonal individuals | 26 | 1994 | 0.190 | 0.190 | -0.0069 (-0.042 to 0.012) |

**Table S4:** Summaries of the linear mixed models used to analyze floral traits of *Daphnopsis filipedunculata* in the FLONA de Carajás, Brazil. npar, number of model parameters; logLik, REM log likelihood; AIC, Akaike information criterion.

| Parameter^1^ | Dropped terms | npar | logLik | AIC | Likelihood ratio test |
| --- | --- | --- | --- | --- | --- |
| Corolla length | none | 4 | -208.3 | 424.6 |  |
|  | floral_type (null model) | 3 | -250.3 | 506.6 | X^2^ = 84.1; df = 1; p < 0.001 |
| Corolla width | none | 4 | -86.2 | 180.4 |  |
|  | floral_type (null model) | 3 | -103.1 | 212.2 | X^2^ = 33.8; df = 1; p < 0.001 |
| Ovary length | none | 4 | -113.7 | 235.4 |  |
|  | floral_type (null model) | 3 | -118.8 | 243.7 | X^2^ = 10.2; df = 1; p = 0.001 |
| Ovary width | none | 4 | 6.4 | -4.8 |  |
|  | floral_type (null model) | 3 | 6.1 | -6.1 | X^2^ = 0.7; df = 1; p = 0.4 |
| Style length | none | 4 | -89.5 | 187.0 |  |
|  | floral_type (null model) | 3 | -92.8 | 191.6 | X^2^ = 6.5; df = 1; p = 0.01 |
| Stigma width | none | 4 | 180.6 | -353.2 |  |
|  | floral_type (null model) | 3 | 143.6 | -281.2 | X^2^ = 74; df = 1; p < 0.001 |

^1^ Full model: Parameter ~ floral_type + (1|repetition). N_obs._ = 159, N_rep._ = 3.


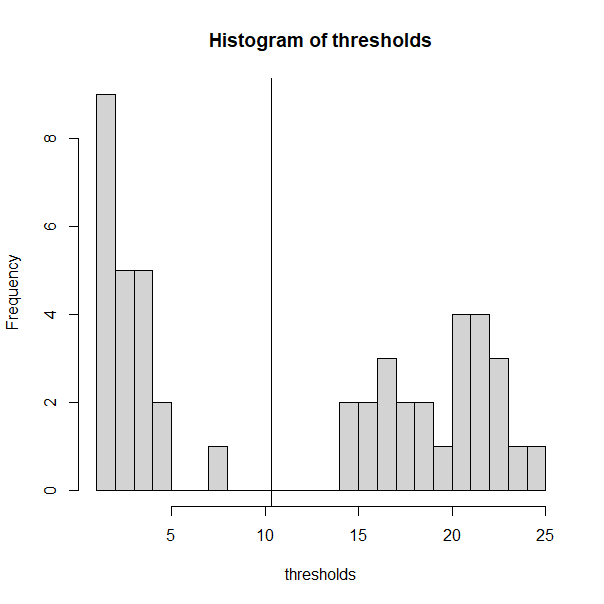


**Figure S1:** Threshold of Euclidean genetic distance (vertical line) among individuals of *Daphnopsis filipedunculata* in the FLONA de Carajás, Brazil. The x-axis and y-axis represent the Euclidean genetic distance and the frequency of pairwise comparison occurrences for a given genetic distance, respectively. The pairwise genetic distance between individuals ranged from 0 to 25, and the genetic assignment threshold was 10.30. Data on the left side of the vertical line represent the genetic variation due to library preparation, sequencing error, and somatic mutations, while data on the right side represent interclonal variation.


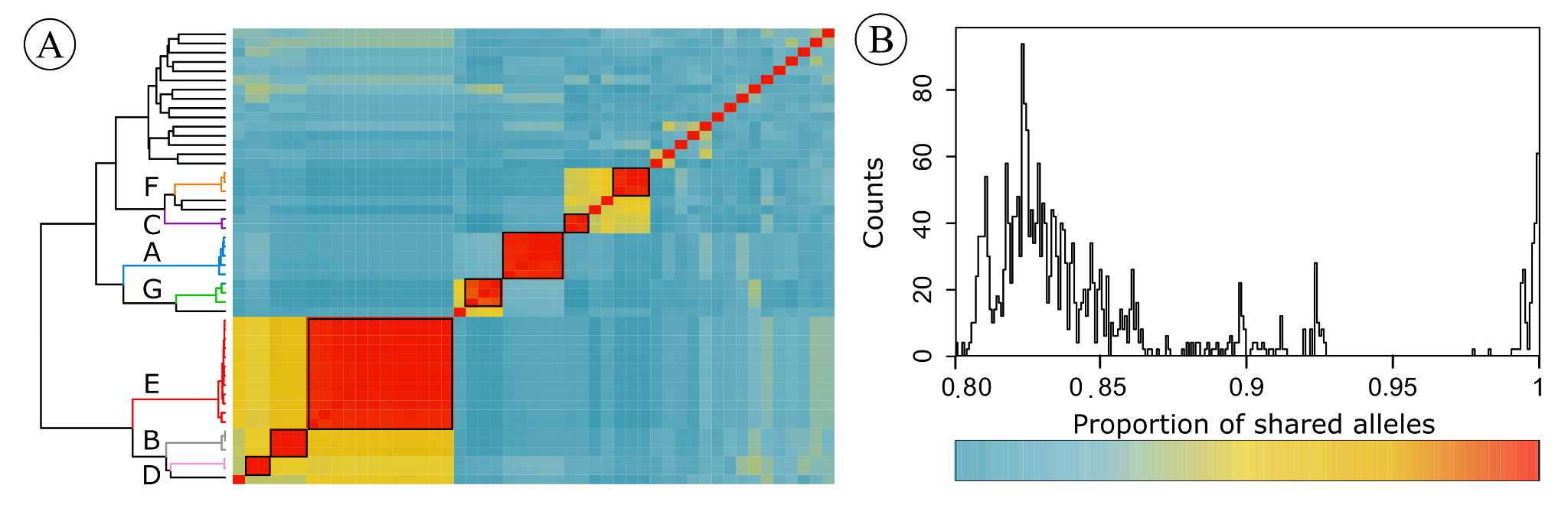


**Figure S2**: Heatmap (A) and histogram (B) showing the proportion of shared alleles between 49 individuals of Daphnopsis filipedunculata. All individuals identified as belonging to same genet (clones) are clustered in the dendogram (A) and share a high proportion of alelles (B). Warmer colors in A represent higher proportion of shared alleles, while colder colors lower proportion of shared alleles between individuals. Letters A to G within the heatmap denote distinct multilocus genotypes. The colors representing each clone in the dendogram in A are according to Fig. 3.

**
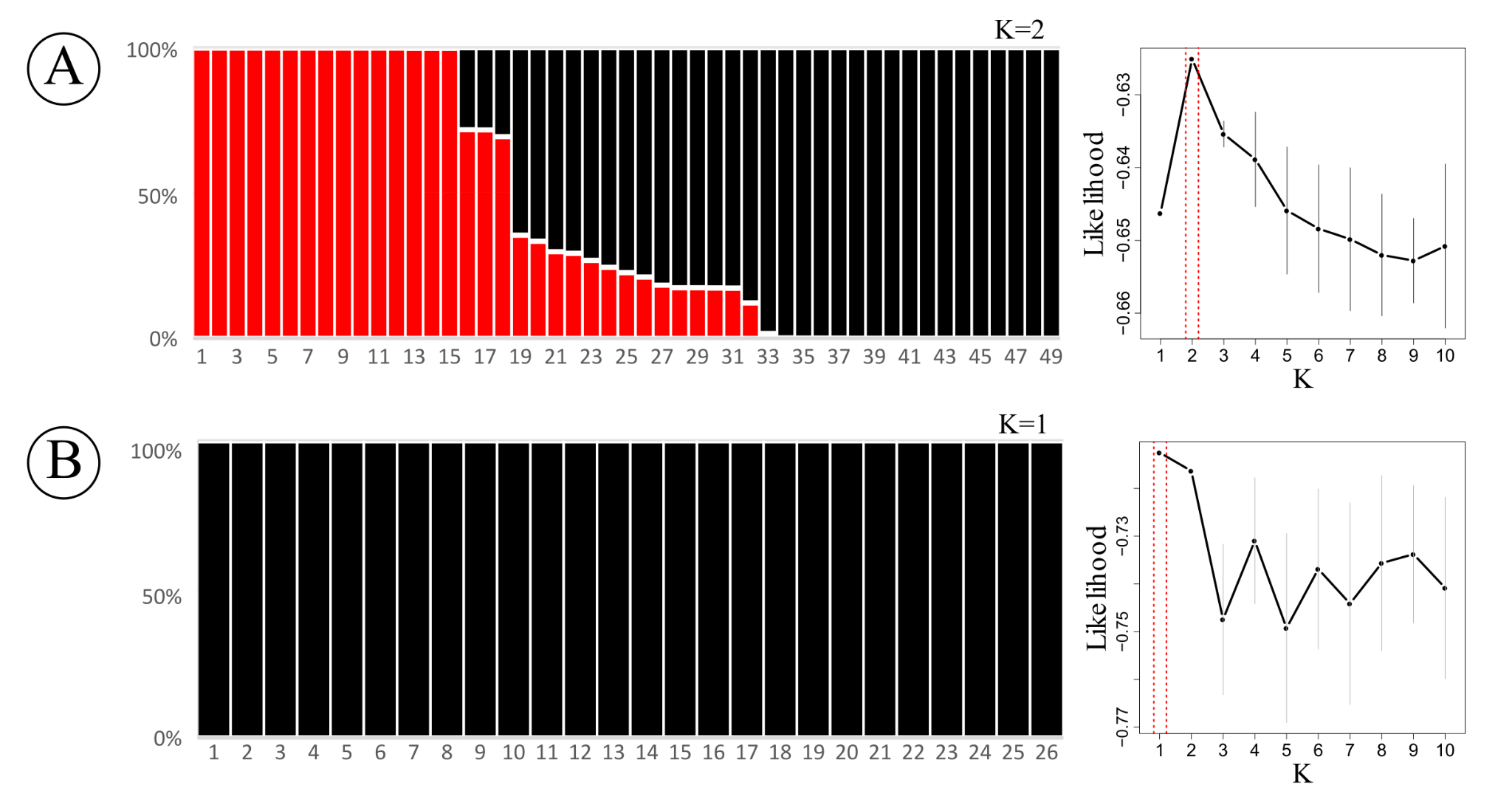
**

**Figure. S3:** Barplot showing fastSTRUCTURE results for the full dataset of 49 clonal and non-clonal individals (A) and a subset of 26 non-clonal individuals of *Daphnopsis filipedunculata* (B), as indicated by the best K (higher likelihood). Each genetic cluster is represented by a different colour in A.
